# Influenza A Co-infection With Atypical Bacteria: A Report of Two Cases

**DOI:** 10.7759/cureus.103459

**Published:** 2026-02-12

**Authors:** Subasima Sekar, Krithika Gopalakrishnan, Sribal Selvarajan, Shanthi Mariappan

**Affiliations:** 1 Department of Microbiology, Sri Ramachandra Institute of Higher Education and Research, Chennai, IND; 2 Department of Microbiology, Sri Ramachandra Institute of Higher Education and Research, chennai, IND

**Keywords:** atypical pneumonia, chlamydia pneumoniae, co-infection, epidemics, illness, influenza a virus, multiplex pcr, mycoplasma pneumoniae

## Abstract

Influenza A virus infection is associated with a wide range of clinical manifestations, from mild upper respiratory infections that resolve on their own to severe pneumonia and sepsis. Bacterial co-infections, particularly due to atypical pathogens such as *Chlamydia pneumoniae* and *Mycoplasma pneumoniae*, may alter disease severity and clinical presentation, especially in paediatric patients and adults with underlying comorbidities. We describe two cases of Influenza A virus infection complicated by atypical bacterial co-infections. The first case involved an elderly female with multiple chronic comorbidities who presented with respiratory sepsis and was found to have co-infection with Influenza A virus and *Chlamydia pneumoniae*. The second patient was a two-year-old female with concomitant infection due to Influenza A virus, *Chlamydia pneumoniae* and *Mycoplasma pneumoniae*. Pathogen identification was achieved using multiplex real-time polymerase chain reaction. In both patients, early initiation of targeted antiviral and antibacterial therapy resulted in clinical improvement and favourable outcomes. Co-infection with the influenza A virus and atypical bacterial pathogens can occur across age groups. Early microbiological diagnosis using multiplex molecular assays supports timely, pathogen-directed therapy and may mitigate disease severity and complications, thereby ensuring the good health and well-being of the patients.

## Introduction

Evidence from the 1918 influenza pandemic first highlighted the critical role of influenza-bacterial co-infections, with secondary bacterial infections being recognized as the likely principal causes of death [1]. Since then, co-infections involving influenza viruses and bacterial pathogens, namely *Staphylococcus aureus, Streptococcus pneumoniae* and *Haemophilus influenzae*, have been well established. These secondary bacterial infections continue to contribute significant morbidity and mortality during seasonal epidemics and pandemics [2].

Apart from these classical pathogens, increasing attention has focused on co-infections between influenza viruses and atypical bacteria, particularly *Mycoplasma pneumoniae* and *Chlamydia pneumoniae* [3]. Accurate etiological diagnosis of such infections remains challenging due to difficulties in obtaining appropriate clinical specimens, distinguishing colonisation from active infection and the limitations of conventional diagnostic methods [4].

*Mycoplasma pneumoniae and Chlamydia pneumoniae* are fastidious pathogens that are difficult to isolate using routine culture techniques, and serological assays frequently produce variable or delayed results, which severely constrain utility in acute clinical settings. Despite the clinical impact of such co-infections, especially in developing countries, their etiological contribution to influenza-associated disease remains under-recognised. For this purpose, molecular diagnostic methods such as polymerase chain reaction provide rapid and reliable diagnosis of atypical bacterial pathogens and their concurrent infection with respiratory viruses, particularly in cases involving patients with lower respiratory tract infections [5].

In this case series, we describe the clinical presentation, diagnostic approach and management, highlighting the importance of recognising viral and atypical bacterial co-infections in patients presenting with respiratory illness. The first case involved an elderly female with multiple chronic comorbidities who presented with acute respiratory symptoms and was found to have a co-infection with Influenza A virus and *Chlamydia pneumoniae*. The second case was a two-year-old female with concomitant infection due to Influenza A virus, *Chlamydia pneumoniae and Mycoplasma pneumoniae.*

## Case presentation

Case 1: Pneumonia due to Influenza A and *Chlamydia pneumoniae* in an adult patient

A 61-year-old woman with a medical history of diabetes mellitus, hypertension and end-stage renal disease, currently receiving maintenance haemodialysis, presented with the complaints of shortness of breath and progressive orthopnoea persisting for three days, as well as low-grade fever (maximum recorded temperature 100.4°F) and dry cough lasting two days. She reported recently attending a public gathering.

**Figure 1:**
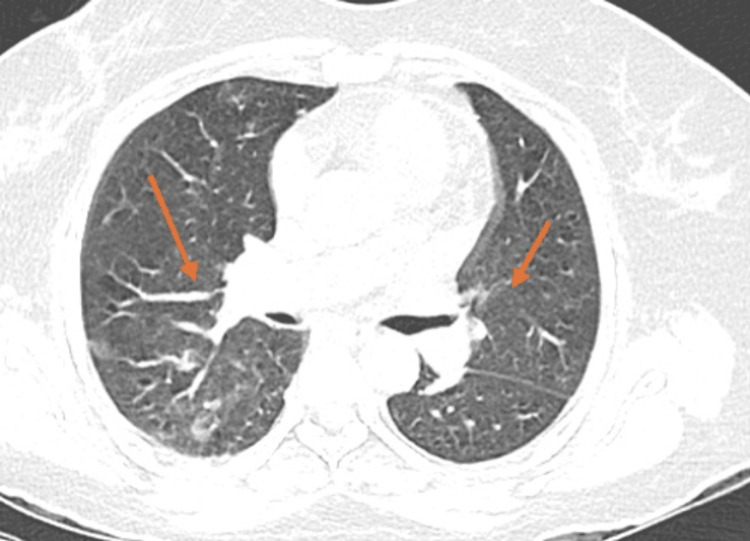
CECT of the thorax. Multiple discrete centrilobular nodules in the bilateral lung. Mild ground glass attenuation is seen in the peribronchovascular location bilaterally. CECT: Contrast-enhanced computed tomography

Upon intensive care unit (ICU) admission, her vital signs were recorded as follows: pulse rate of 92 beats per minute, respiratory rate of 36 per minute, blood pressure at 130/70 mmHg and oxygen saturation (SpO₂) at 97%. A physical examination showed bilateral crepitations upon chest auscultation. Both cardiovascular and neurological assessments were unremarkable. Laboratory tests revealed anaemia, normal total leukocyte count, high polymorphs, mild thrombocytopenia and elevated C-Reactive Protein (Table 1). Radiological evidence was suggestive of atypical pneumonia (Figure 1).

**Table 1:** Laboratory investigations

Laboratory tests	Case 1	Case 2	Normal range
Haemoglobin (g/dL)	6.2	11.8	Adult: 12 - 15; Paediatrics: 11 - 14
Total leukocyte count (cells/mm³)	9810	8020	Adult: 4,000 - 11,000; Paediatrics: 5000 - 17000
Platelet count (lakhs/mm³)	1.47	2.93	Adult: 1.5 - 4.5; Paediatrics: 2 - 4.9
Polymorphs (%)	82.5	60.4	Adult: 45 - 70; Paediatrics: 30 - 40
Lymphocytes (%)	12.5	34.5	Adult: 25 - 40; Paediatrics: 25 - 45
Eosinophils (%)	0.1	0.4	1 - 6
Monocytes (%)	4.0	4.2	2 - 10
Basophils (%)	0.1	0.1	0 - 1
C-Reactive Protein (mg/dl)	2.4	1.2	Positive - > 0.8; Negative - < 0.8

She was initially managed for suspected volume overload with diuretics and non-invasive ventilation. Throat and nasal swabs were collected for testing of respiratory pathogens using real-time polymerase chain reaction, which detected Influenza A virus and *Chlamydia pneumoniae*. Empirical intravenous amoxicillin-clavulanate was initiated on admission. Following PCR confirmation of Influenza A with *Chlamydia pneumoniae* co-infection, antimicrobial therapy was de-escalated to intravenous azithromycin and oral oseltamivir. Azithromycin was given for four days, then switched to oral doxycycline to complete a seven-day course, while oseltamivir was continued for five days. After clinical stabilization, the patient was gradually weaned off non-invasive ventilation, transferred to a step-down unit and subsequently discharged upon complete resolution of symptoms.

Case 2: Pneumonia due to Influenza A, *Chlamydia pneumoniae* and *Mycoplasma pneumoniae* 

A two-year-old female presented with a six-day fever history (maximum recorded temperature 103°F), cough, coryza, ear pain, reduced activity and decreased oral intake. There was a past history of simple febrile seizures at 1.5 years of age. Developmental milestones were appropriate for age, and the child had received all age-appropriate immunisations.

On examination, the child was mildly lethargic, had a pulse rate of 124 beats per minute, a respiratory rate of 30 breaths per minute, blood pressure measuring 80/60 mmHg and an oxygen saturation level of 98% on room air. The overall systemic examination yielded no significant findings.

**Figure 2:**
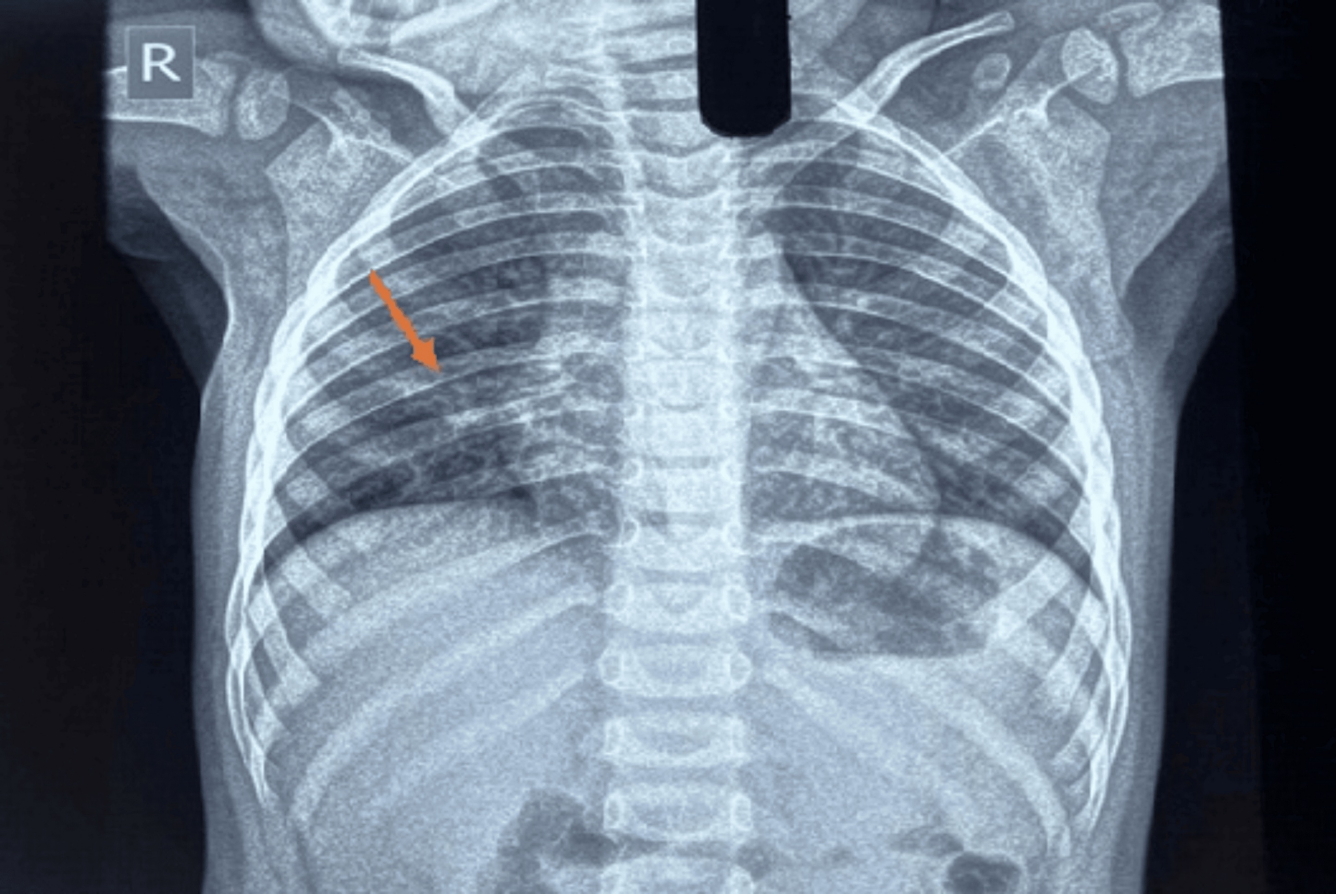
Chest X-ray Bilateral patchy perihilar and lower-zone air-space opacities with prominent bronchovascular markings suggestive of pneumonitis.

Laboratory investigations revealed elevated C-Reactive Protein levels, while the complete blood counts were within normal limits (Table 1). Radiological evidence was suggestive of atypical pneumonia (Figure 2).

Real-time PCR testing of throat and nasal swabs was positive for Influenza A virus, *Chlamydia pneumoniae and Mycoplasma pneumoniae*. The patient received treatment with oral oseltamivir 2.5 mL twice daily for three days, oral azithromycin 3.5 mL once daily for five days, levocetirizine 2.5 mL twice daily for five days and paracetamol 250 mg as needed. Blood cultures were sterile. The child’s oral intake improved, and she remained afebrile for more than 12 hours, following which she was discharged in stable condition.

## Discussion

Influenza A virus infection is a major cause of acute respiratory illness and is associated with significant morbidity and mortality, particularly when complicated by bacterial co-infections. Polymicrobial infections involving two or more pathogens occur in 5-40% of cases. Influenza is characterised by rapid clinical onset and marked seasonal variation. Approximately 10% of the global population contracts an infection each year, accounting for an estimated 500,000 deaths each year [6]. Transmission occurs via respiratory droplets, contact and aerosols, and the virus is capable of prolonged survival under favourable environmental conditions [7].

While classical bacterial pathogens such as *Staphylococcus aureus, Streptococcus pneumoniae* and *Haemophilus influenzae* are well-recognised contributors to severe influenza-associated disease, reports regarding atypical bacterial pathogens are limited. *Mycoplasma pneumoniae* and *Chlamydia pneumoniae* are recognized causes of atypical community-acquired pneumonia (CAP) but are likely under-recognised due to diagnostic limitations. *Chlamydia pneumoniae* accounts for approximately 10% of CAP cases and affects all age groups, particularly school-aged children and older adults. Reinfection is common throughout life and is more frequently detected during colder months [4,7]. *Mycoplasma pneumoniae* accounts for 32.4%-39.5% of paediatric CAP cases and predominantly affects school-aged children and young adults (5-20 years), where it typically causes subacute atypical pneumonia. It is less common in children under five years and exhibits cyclical outbreaks every three to seven years, with seasonal predominance in late summer and autumn [8]. Although dual infections with the influenza A virus and either *Mycoplasma pneumoniae or Chlamydia pneumoniae* have been reported, documented triple infections involving all three pathogens are rare. The identification of such co-infections in this series suggests that their true incidence may be underestimated due to limitations of conventional diagnostic methods.

In Case 1, CECT of the thorax revealed bilateral centrilobular nodules with peribronchovascular ground-glass opacities, while in Case 2, the chest X-ray showed bilateral patchy perihilar and lower-zone air-space opacities with prominent bronchovascular markings. The radiological appearances in both cases were consistent with atypical pneumonia.

Influenza virus infection increases susceptibility to bacterial superinfections by causing epithelial damage, impairing mucociliary clearance and altering immune responses [9]. Such bacterial superinfections, including those caused by atypical bacteria, can exacerbate respiratory compromise, particularly in patients with comorbidities or immature immune systems. The mechanisms responsible for the co-infection of influenza virus with *Mycoplasma pneumoniae or Chlamydia pneumoniae* have not been directly studied. Immune responses to *Mycoplasma pneumoniae and Chlamydia pneumoniae* are largely mediated through Toll-like receptor 2 (TLR-2), which is also important for effective clearance of the influenza virus. Activation of shared TLR-2-dependent pathways may therefore limit influenza coinfection. However, this antagonistic relationship may be explained by differences in innate immune activation [3,7]. Coinfection with atypical pathogens can result in extended viral persistence, increased infiltration of immune cells in the lungs and markedly higher concentrations of inflammatory cytokines (such as IL-6, CCL3, CCL4 and G-CSF), ultimately leading to severe pneumonia [10].

Conventional microbiological methods are limited for *Mycoplasma pneumoniae and Chlamydia pneumoniae*, as these organisms are fastidious and not readily isolated by routine culture, while serological assays are constrained by delayed antibody responses and variable performance [11]. In this context, simultaneous detection of Influenza A virus and atypical bacterial pathogens from respiratory tract specimens using real-time PCR provided timely results, directly enabling the early initiation of pathogen-directed therapy. In both patients, early targeted antiviral and antibacterial therapy was associated with favourable clinical outcomes, with no progression to respiratory failure, prolonged hospitalisation or mortality. The consistent clinicomicrobiological correlation observed supports the pathogenic relevance of the detected organisms.

Establishing a specific diagnosis of atypical bacterial co-infection has direct therapeutic significance. *Mycoplasma pneumoniae and Chlamydia pneumoniae* either lack a typical peptidoglycan cell wall or have intracellular growth requirements, rendering β-lactam antibiotics, which are commonly used as first-line empirical therapy for CAP, ineffective against these pathogens [4,7]. Failure to recognise these organisms may therefore result in inappropriate antimicrobial therapy and delayed clinical response. Accurate identification of these atypical pathogens is essential to guide the selection of appropriate agents such as macrolides, tetracyclines or fluoroquinolones, thereby improving treatment efficacy and supporting antimicrobial stewardship.

## Conclusions

This case series highlights that Influenza A virus infection may be complicated by co-infection with atypical bacterial pathogens. Such co-infections contribute to increased disease severity and pose significant diagnostic and therapeutic challenges. The use of molecular diagnostic techniques allows rapid and sensitive detection of viral and atypical bacterial pathogens from respiratory specimens.

Early and specific pathogen identification is crucial not only for optimal clinical management but also to avoid ineffective β-lactam therapy in atypical pneumonia, reduce unnecessary broad-spectrum antibiotic use, and strengthen antimicrobial stewardship. Overall, this diagnostic approach enhances clinical decision-making and is associated with favourable patient outcomes.
